# Validity, Reliability and Sensitivity to Change of Three Consumer-Grade Activity Trackers in Controlled and Free-Living Conditions among Older Adults

**DOI:** 10.3390/s21186245

**Published:** 2021-09-17

**Authors:** Kaja Kastelic, Marina Dobnik, Stefan Löfler, Christian Hofer, Nejc Šarabon

**Affiliations:** 1 Andrej Marušič Institute, University of Primorska, Muzejski Trg 2, 6000 Koper, Slovenia; kaja.kastelic@iam.upr.si; 2 InnoRenew CoE, Livade 6, 6310 Izola, Slovenia; 3 Faculty of Health Sciences, University of Primorska, Polje 42, 6310 Izola, Slovenia; marina.dobnik@fvz.upr.si; 4 Physiko-& Rheumatherapie, Institute for Physical Medicine and Rehabilitation, Neugebäudeplatz 1, 3100 St. Pölten, Austria; stefan.loefler@kern-reha.at; 5 Ludwig Boltzmann Institute for Rehabilitation Research, Neugebäudeplatz 1, 3100 St. Pölten, Austria; christian.hofer@rehabilitation.lbg.ac.at; 6 Centre of Active Ageing—Competence Centre for Health, Prevention and Active Ageing, Neugebäudeplatz 1, 3100 St. Pölten, Austria

**Keywords:** healthy aging, fitness tracker, daily activity behaviors, physical behaviors, physical activity, sedentary behavior, sleep, responsiveness, comparison, mHealth

## Abstract

Wrist-worn consumer-grade activity trackers are popular devices, developed mainly for personal use. This study aimed to explore the validity, reliability and sensitivity to change of movement behaviors metrics from three activity trackers (Polar Vantage M, Garmin Vivoactive 4s and Garmin Vivosport) in controlled and free-living conditions when worn by older adults. Participants (n = 28; 74 ± 5 years) underwent a videotaped laboratory protocol while wearing all three trackers. On a separate occasion, participants (n = 17 for each of the trackers) wore one (randomly assigned) tracker and a research-grade activity monitor ActiGraph wGT3X-BT simultaneously for six consecutive days. Both Garmin trackers showed excellent performance for step counts, with a mean absolute percentage error (MAPE) below 20% and intraclass correlation coefficient (ICC_2,1_) above 0.90 (*p* < 0.05). The MAPE for sleep time was within 10% for all the trackers tested, while it was far beyond 20% for all other movement behaviors metrics. The results suggested that all three trackers could be used for measuring sleep time with a high level of accuracy, and both Garmin trackers could also be used for step counts. All other output metrics should be used with caution. The results provided in this study could be used to guide choice on activity trackers aiming for different purposes—individual use, longitudinal monitoring or in clinical trial setting.

## 1. Introduction

Measurement of health-related movement behaviors (e.g., physical activity (PA), sedentary behavior (SB), sleep) is fundamental for research and practice [1]. Historically, only self-reported questionnaires that come with the recall and social desirability bias were available [2]. In order to overcome those limitations and to further develop the field, wearable devices that measure free-living movement behaviors were introduced. The most frequently used technology that those activity monitors rely on is the accelerometry [3]. In some devices it is also accompanied by other sensors, like heart rate (HR), galvanic skin response and/or skin temperature sensors, etc. [4,5].

Technological development led to miniaturizing devices, expanding the battery life, computing diverse movement behaviors and computing vital signs metrics, while becoming affordable for the use in large-scale studies [6], clinical applications [7] or for individual consumers. Specifically, wrist-worn consumer-based activity trackers gained popularity in recent decades. The market of wearable devices is growing rapidly; there were 722 million connected devices in 2019, and it has been forecasted that a billion will be reached in 2022 [8]. Despite that wrist-worn trackers were mainly designed for motivating consumers to increase their PA participation [9,10], the devices are also attractive for research and clinical purposes due to high compliance and relatively low cost. They were designed to be user friendly and suitable for long-term use, which makes them especially attractive for long-term clinical applications.

New models of consumer-based activity trackers and new algorithms that compute already known and new and innovative movement behaviors metrics are released regularly. The latter is the reason why exploring the measurement properties of the computed metrics is a continuous task. Only metrics that are reasonably reliable, valid and/or sensitive to detect changes are valuable for research or practice. Moreover, knowing measurement properties of metrics from different measurement tools is important to be able to select a proper tool for a specific situation [11,12].

The choice of the measurement tool and the time frame captured should be guided by the purpose of the measurement [12]. For example, if the adherence to PA guideline on the individual level is of interest, then the selected tool needs to provide a highly accurate and precise measure of PA. However, if adherence on the group or population level is of interest, then lower precision is also acceptable. Furthermore, if the purpose of the measurement is to address changes in the behavior, then the sensitivity to detect changes is of great importance [12]. Specifically, when changes on the individual level are of interest, then the minimal detectable change (MDC; e.g., change in behavior that is beyond the within-subject variability of behavior and the measurement error) of the metric should be low, but when the changes on the group or population level are of interest, the responsiveness of the metric needs to be high [12]. To date, the sensitivity to detect changes has received little attention in the validation studies of the activity trackers [13].

Considerable effort has already been made in validating the output metrics from the consumer-based activity trackers [14,15,16]. The performance of the activity trackers differs substantially between different brands and models, as well as between the populations tested. In general, the accuracy of step counts is high in the adult population without mobility limitations, while validity for measuring PA, energy expenditure and sleep ranges from poor to good. Most studies were performed in controlled conditions and step counts were the prevailing outcome metric [16]. Exploring the validity of the specific output metric while performing a structured protocol of selected movement activities in a laboratory environment offers an insight into activity-specific challenges for accurate capturing [17]. However, such findings have only limited value when one is interested in the performance of the activity tracker in the free-living environment. To date, substantially more validation studies included healthy younger and middle-aged adults than older adults [14,16]. There is a need for validation studies among older adults, since the performance of the activity trackers might differ between younger and older adults (e.g., due to differences in gait patterns that might affect step counting performance, and due to differences in patterns of PA accumulation and skin perfusion that might affect PA intensity classification performance).

Therefore, the aim of this study was to test the validity, reliability and sensitivity to change of several movement behavior metrics from three consumer-based wrist-worn activity trackers (Polar Vantage M, Garmin Vivoactive 4s and Garmin Vivosport) in a population of healthy older adults. We chose to test those specific three activity trackers because they are popular wearables that have never been validated in the older adult population and due to our institutional access to those specific devices. We hypothesized that the accuracy of the step counts would be high, while it would be modest for all other movement behaviors metrics.

## 2. Materials and Methods

### 2.1. Participants Recruitment

A convenience sample of older adults from Central and Western regions of Slovenia were included in the study. Participants were recruited via several centers of daily activities for older adults. The inclusion criteria were an age of 60 years or above, able to walk independently without mobility aids (e.g., a cane or rollator) and the absence of substantial (self-reported) neurological and cognitive impairments. The study was approved by the Republic of Slovenia’s National Medical Ethics Committee (approval number: 0120-321/2017-4) and was performed in line with Helsinki declaration. Participants received verbal explanation of the protocol and the opportunity to ask questions before they signed the written informed consent to participate in the study.

**Figure 1:**
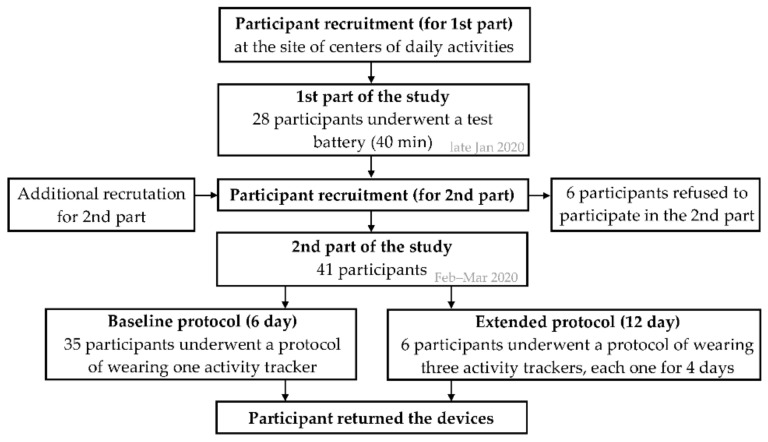
A flowchart of the study activities.

The study consisted of two parts: the first part was conducted in a laboratory environment, where only step counts were of interest; and the second part was conducted under free-living conditions, where steps, active calories burned, movement behavior and sleep metrics were of interest (Figure 1). According to the published recommendations for determining the validity of consumer-based activity trackers, a sample size of a minimum of 15 participants is required for validation studies including only older adults [18].

In the first part of the study, 28 older adults (15 female) from 67 to 84 years of age successfully completed the testing (all but two were right-handed participants). In the second part of the study, 41 participants (25 female) from 60 to 84 years of age were included (all but one were right-handed participants); 35 participants underwent the protocol of wearing one randomly assigned activity tracker for six days, and 6 participants wore all three activity trackers, each one for four days (for detailed information see the study protocol subsections). In total, 53 participant-device (free-living) measurements were taken, of which 50 were considered valid and were included into the further analysis. All participants were generally healthy, able to walk independently without mobility aids, free of substantial neurological and cognitive impairments and lived in a private home setting. All participants had white-colored skin. The participant’s characteristics are shown in Table 1.

### 2.2. Measurement Devices and Equipment

Three consumer-grade activity trackers (Polar Vantage M, Garmin Vivoactive 4s, Garmin Vivosport) were tested in controlled and free-living conditions. A video camera (Panasonic HDC-HS900) and research-grade accelerometer (ActiGraph wGT3X-BT) were used as a criterion and convergent measure in controlled and free-living conditions, respectively.

- Polar Vantage M (Polar Electro OY, Kempele, Finland) is a multi-sensor wrist-worn activity tracking device (mass 45 g) that computes data on the following: steps taken, calories burned, distance travelled and time spent resting (sleep and rest, lying down) and sitting (sitting or other passive behavior); time spent in low-intensity activity (standing work, light household chores), medium-intensity activity (walking and other moderate activities) and high-intensity activity (jogging, running and other intense activities); and some others. The activity tracker is water resistant with a battery autonomy of up to 7 days (suggested price: EUR 279.95, on-the-market date: October 2018).
- Garmin Vivoactive 4s (Garmin, Olathe, Kansas, USA) is a multi-sensor wrist-worn activity tracking device (mass 40 g) that computes data on steps taken, calories burned, floors climbed, distance travelled, intensity minutes (i.e., minutes of moderate-to-vigorous physical activity (MVPA) in bouts of at least 10 min, where vigorous minutes are doubled when added), total sleep time and some others. The activity tracker is water resistant with a battery life of up to 7 days (suggested price: EUR 279.99, on-the-market date: October 2019).
- Garmin Vivosport (Garmin, Olathe, KS, USA) is a multi-sensor wrist-worn activity tracking device (mass 27 g) that computes data on steps taken, calories burned, floors climbed, distance travelled, intensity minutes, total sleep time and some others. The activity tracker is water resistant with a battery life of up to 7 days (suggested price: EUR 109.99, on-the-market date: September 2017).
- Panasonic HDC-HS900 (Panasonic Corporation, Kadoma, Osaka, Japan) is a high-resolution video camera, with effective video resolution of 2.53 MP, a focal length of 3.45–41.4 mm and automatic or manual focus adjustments. The digital video format is AVCHS 1920 × 1080, and the camera stores data on the 220 GB HDD internal storage or external SD memory card.
- ActiGraph wGT3X-BT (ActiGraph LLC, Pensacola, FL, USA) is small and light (4.6 cm × 3.3 cm × 1.5 cm; 19 g) research-grade physical activity monitor (3-axial accelerometer), which provides a variety of physical activity and sleep measures over the 24-hour movement continuum (sleep time and sedentary time; light-, moderate- and vigorous-intensity physical activity; as well as bout length of each activity, steps taken, activity counts, energy expenditure and some others). Sampling frequency can be set manually from 30 to 100 Hz. The device is not fully water resistant (i.e., not made for swimming) with a battery life of up to 28 days.

### 2.3. Study Protocol in Controlled Conditions

**Figure 2:**
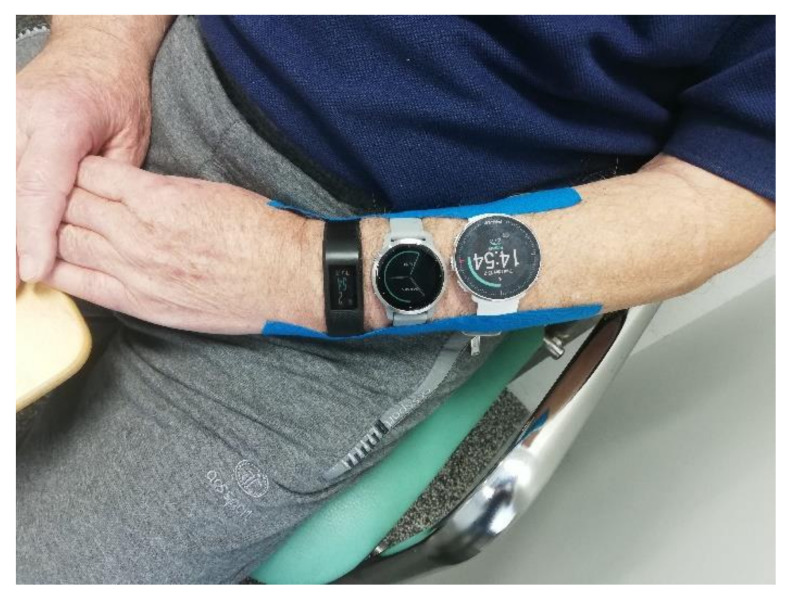
Participants wore all three activity monitors on their non-dominant wrist during testing in controlled conditions. The devices were additionally taped to the participant’s forearm to ensure they stayed in a fixed position. The order (proximal to distal) was the same for all participants: the Polar Vantage M, Garmin Vivoactive 4s and Garmin Vivosport.

The first part of this study was performed in a controlled laboratory setting (air-conditioned, non-obstacle space 8 × 7 m). Participants underwent a test battery that included common real-life tasks (sedentary activity, walking and household activity), while wearing all three activity trackers on their non-dominant wrist. The activity trackers were additionally fixed using adhesive tape, to assure they stayed in position while participants underwent the testing (Figure 2). Before the testing, a researcher explained the study protocol and demonstrated each task. The test battery consisted of four tasks, each followed by a 5 min seated break:

- Preferred pace walking (for 5 min);
- Slow pace walking (for 5 min);
- Tidying the dish (for 5 min);
- Playing cards task (for 5 min).

The order of the tasks was randomized, but only the combinations where preferred pace walking came prior to slow-pace walking were included (because walking pace during the slow pace task was calculated based on the individual’s preferred pace). The participants were asked to perform the tasks as they would in real-life situations. For the walking tasks, participants were instructed to walk evenly and follow the ellipse line on the floor (with a circumference of 15.1 m). They were asked to change the direction of walking after 2.5 min, or before, if they started feeling dizzy. For the preferred pace walking task, they were instructed to walk normally at their own pace. The researcher calculated the participants’ average time needed for one lap during preferred pace walking and added 30% of the time, to calculate the targeted time needed for a slow pace walking task (a similar approach could be found elsewhere [19]). For slow pace walking, they were instructed to walk slowly. Then the researcher measured the time needed for one lap and verbally directed them to slow down or speed up, in order to achieve the targeted pace. For the playing cards task, the participant was instructed to mix the cards (The Donkey) for 10 cycles, distribute the cards to two players and then play the game with the researcher. If the game was over before 5 min passed, the participant was asked to repeat the game. For tidying the dish task, the participant was instructed to get the dish from the cupboard to the working surface, then wipe it with the cloth and put it back to the cupboard at their preferred pace. If the task was over before 5 min passed, then the participant was asked to repeat the task. The researcher instructed verbally when a specific task was done, asking the participant to sit on a chair for 5 min.

In this part of the study, only the step counts were of interest. The researcher transcribed the number of steps from each of the three activity trackers tested prior to the first task and then after each task was done. Since we noticed that some activity trackers needed some time to catch up with the actual steps, we transcribed the steps after 2.5 min of a seated break (the participants were instructed not to move their arms during the seated breaks). During the visit, the participants were videotaped continuously (using two video cameras of Panasonic HDC-HS900), allowing the researcher to document each step taken and count it visually afterwards.

### 2.4. Study Protocol in Free-Living Conditions

The second part of this study was performed in free-living conditions. Participants were asked whether they are prepared to participate in a study for 6 days (baseline protocol) or for 12 days (extended protocol). Those who had consented to participate in a baseline protocol were encouraged to wear one (randomly assigned) consumer-based activity tracker on their non-dominant wrist (on top of the wrist, at least a finger’s width up from the wrist bone), and a research-grade physical activity monitor, the ActiGraph, on their waist over the dominant leg for 6 days simultaneously (24 h per day). Those who had consented to participate in the extended protocol wore all three consumer-based activity trackers, each one for four days (simultaneously with ActiGraph). They were instructed to remove the ActiGraph only for showering or swimming. The participants received verbal instructions and practical demonstration on how to allocate and wear the devices (in addition to written instructions and a telephone number of a researcher to call, if any questions would arise during the study). Participants were also asked to keep a sleep and wake diary (on paper), and not to enter into the activity trackers’ menus and make any changes. During the study, all notifications on the activity trackers were turned off, and only the date and time information was visible on the display.

Before a specific activity tracker was given to each participant, all the previously stored data in the device were deleted and the required personal information of the participant (e.g., sex, age, body weight, body height) were entered into the device. Additionally, we created a new user account for each participant in order to avoid any miscalculations—the underlying algorithms of activity trackers are not available, but it can be found in the manufacturer’s manuals that some calculations rely on previously stored information. For example, Garmin’s algorithm that computes daily intensity minutes includes the average resting heart rate, which can be stored from the previous user if memory is not deleted.

### 2.5. Data Management and Outcome Measures

For the part of the study in controlled conditions, transcribed data of the number of steps shown by the activity trackers were entered into the Excel file. Then the differences between adjacent transcriptions were calculated to obtain the number of steps counted during each 5 min task for each activity tracker. Watching the videos, the researcher visually counted the number of actual steps taken during each 5 min task and entered it into the Excel file. This method is considered to be a gold standard for counting steps [20] and was chosen as a criterion measure in this part of the study.

During the second part of the study in the free-living environment, data were collected using an activity tracker and the ActiGraph simultaneously. The ActiGraph (with self-reported sleep time information) was considered as a convergent measure. Self-reported sleep and wake times from the diaries were transcribed into the Excel file. We defined a valid day if there were ≥13 awake hours of wear [21], using the midnight-to-midnight approach (only valid days were included in the further analysis). All activity trackers used the most up-to-date firmware at the time the study was conducted (Polar Vantage M software version 5.0.10; Vivoactive 4s software version 4.70; Vivosport software version 4.20). Data from the Polar devices were transferred via the Polar FlowSync application (Version 3.0.0.1337) and from the Garmin devices via the Garmin Express application (Version 7.0.1.0). The computed outputs from both Garmin devices were downloaded as .csv files. The time of detected sleep onset and offset was captured from the application window and manually transcribed into the Excel file (because downloads from the application were not possible). For the same reason, the computed outputs from the Polar device were also captured from the application and manually transcribed. It is worth noting that manufacturers’ algorithms to compute variables are not available, nor are the raw data or sampling frequency. Activity trackers used were brand new at the start of our study.

The outcome measures of the Polar Vantage M were steps, calories burned and time spent resting, sitting and in low-, medium- and high-intensity activity, as well as the difference between the times of sleep onset and offset. The polar device computed total calories burned, so we calculated the basal metabolic rate (BMR) using the widely adopted equation from Harris and Benedict [22] and the deducted BMR from total calories to obtain active calories only. Since the time spent resting also includes sleep time, we subtracted sleep time and added that difference to sitting time in order to obtain sedentary time (which is defined as sitting or lying while awake with low metabolic consumption [23]). The outcome measures of interest for both Garmin activity trackers (Vivoactive 4s and Vivosport) were steps, intensity minutes, active calories burned and the difference between the time of sleep onset and offset. We determined the Polar and Garmin wear time via visual inspection of heart rate (HR) data (similar approach could be found elsewhere [24]). Wear time was considered for periods where the HR was available.

Self-reported sleep time was used as a convergent measure for sleep time, while ActiGraph data were used for all other metrics. Data from the ActiGraph were downloaded and processed using ActiLife software (ActiLife v6.13.3, ActiGraph Corp., Pensacola, FL, USA). Sampling frequency was set at 30 Hz, and data were captured in 60 s epochs. For wear time validation, we used Choi et al.’s [25] algorithm. Only the reported periods of wake time were validated and included into the further analysis. To determine the time spent in each movement behavior, we applied activity cut points by Troiano et al. [26]. Sedentary behavior was defined as 0 to 99 counts per min (cpm), light activity as 100 to 2019 cpm, moderate activity as 2020 to 5998 cpm and vigorous activity as 5999 cpm and above. Additionally, we calculated the bouted MVPA, which was defined as 2020 cpm and above in at least 10 min bouts, with an allowance of 1 or 2 min below the threshold [26]. Energy expenditure was computed using the Freedson VM3 Combination 2011 algorithm [27].

### 2.6. Statistical Analysis

Statistical analyses were performed using Microsoft Excel and SPSS, version 18.0 (SPSS Inc., Chicago, Ill., USA). The analytic methods to evaluate the validity of the activity monitors followed the previously published recommendations [17]. The comparisons between the computed measures from activity trackers and the criterion/convergent measures were done using the error indicators, intraclass correlation coefficient (ICC) and the Bland–Altman method. The mean error (ME) and mean percentage error (MPE) were calculated to explore group-level agreement, and the mean absolute percentage error (MAPE) and root mean square error (RMSE) were used to explore agreement on an individual level (for the error indicators, tested values were deducted from the criterion/convergent values). The two-way mixed model ICC_2,1_ was used to measure the extent of the agreement between the activity tracker and the criterion/convergent measure. The related 95% confidence intervals were also calculated. An ICC ≥ 0.75 was considered excellent, 0.60–0.74 was good, 0.40–0.59 was fair and <0.40 was poor [28]. Parameters for constructing Bland–Altman plots were calculated to explore the systematic and random error. The Shapiro–Wilk test was used to test for normal distribution of mean error and then the paired sample t-test was applied to test for systematic difference (i.e., bias). Statistical significance was set at α < 0.05.

Additionally, absolute and relative reliability, minimal detectable change and responsiveness to change for single-day measures and for three-day average measures were also tested [12]. First, a single factor within-subjects analysis of variance (ANOVA) was used to test the differences between the multiple days of monitoring and the F-test to evaluate the presence of systematic error. The partial eta squared (η_p_^2^) was calculated to examine the effect size of the ANOVA. Second, the relative reliability was tested using the intraclass correlation coefficients (ICC_2,1_) for single-day measures and for three-day average measures. Third, absolute reliability was tested using standard error of measurement (SEM). Fourth, minimal detectable changes (MDC) were calculated using a formula: MDC = SEM × 1.96 × √2. The MDC indicates a change in the individual level, which can be interpreted as real change (that is beyond the normal within-subject day-to-day variability in behaviors and the measurement error). Fifth, to examine the responsiveness to change (ie, ability to detect changes on a group level) of single-day measures (two randomly selected days were included) and of three-day average measures (average of the first three days and second three days), the Cohen’s effect size (ES) and Guyatt’s responsiveness coefficient (GR) were calculated. The detailed description of the tests used can be found elsewhere [12,29].

## 3. Results

### 3.1. The First Part: Controlled Conditions

**Figure 3:**
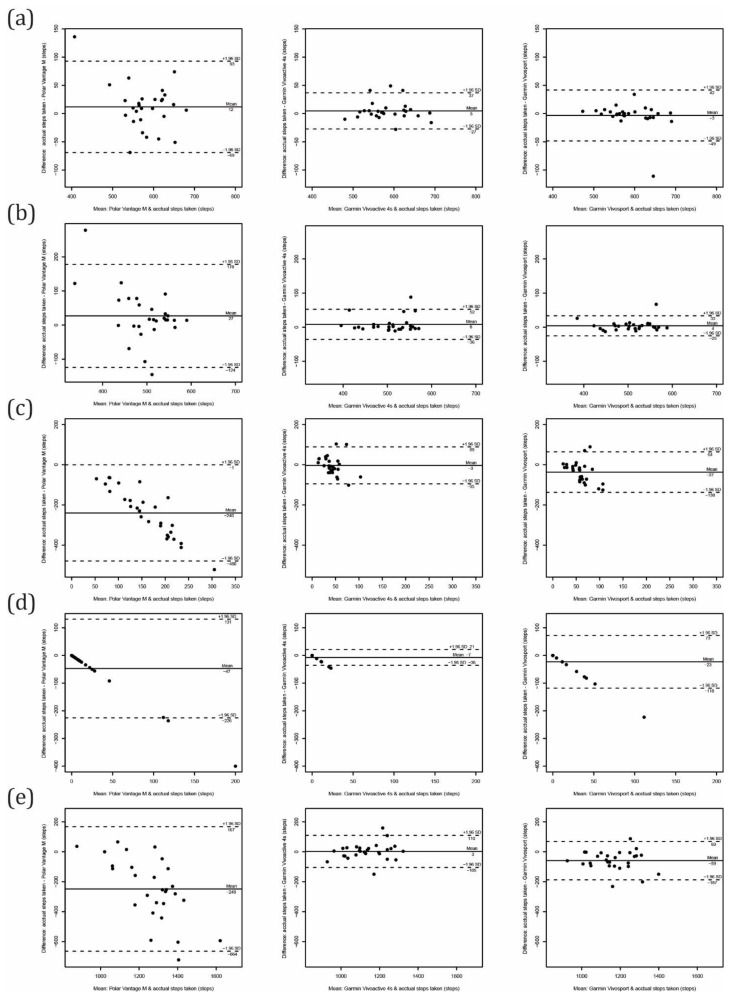
Bland–Altman plots for controlled conditions tasks: (**a**) preferred pace walking, (**b**) slow pace walking, (**c**) tidying the dish, (**d**) playing cards and (**e**) all controlled tasks.

The step-counting performance for all activity trackers/tasks performed in a controlled condition is presented in Table 2 and Figure 3. The average calculated walking speed for preferred pace walking was 4.4 ± 0.6 km/h and for slow pace walking was 3.4 ± 0.4 km/h. All activity trackers showed their best performance for the preferred pace walking task and slightly lower performance for the slow pace walking task. The MAPE was as low as 2% for both walking tasks for both the Garmin devices (Vivoactive 4s and Vivosport), and the ICC_2,1_ was also excellent. The MAPEs for the Polar Vantage M were 6% for preferred walking and 11% for slow walking, while ICC_2,1_ values were excellent and poor, respectively.

For tidying the dish task, the performance of all activity trackers was poor (ICC_2,1_ ranging from −0.18 to −0.00). The t-test revealed a systematic error for the Polar Vantage M and Garmin Vivosport, which overestimated steps. The MAPE exceeded 100% for all devices, with the highest MAPE for the Polar Vantage M (751%). During the 5 min task, an overestimation of 240 ± 122 steps was observed for the Polar Vantage M. Similarly, for playing the cards task, the Polar Vantage M (over)counted 47 ± 91 steps, followed by the Garmin Vivosport (23 ± 49 steps) and Garmin Vivoactive 4s (7 ± 14 steps).

Comparing all steps counted during the testing protocol (that lasted for 40 min), the Polar Vantage M and Garmin Vivosport significantly overestimated steps taken; for 248 ± 212 and 59 ± 65 steps with a MPE of 22% and 5%, respectively. The overall best performance was observed for the Garmin Vivoactive 4s, with a MPE of 0% and MAPE of only 3%. The ICC_2,1_ also revealed excellent agreement with the criterion measure.

### 3.2. The Second Part: Free-Living Conditions

According to the ActiGraph (and self-reported sleep time information), which served as a convergent measure, our sample of older adults spent on average 10.4 (±1.9) h/day, 5.0 (±1.4) h/day and 45 (±36) min/day in sedentary behavior, light-intensity physical activity (LIPA) and MVPA, respectively, and took 8446 (±3774) steps/day. They engaged in bouted MVPA (i.e., MVPA that was accumulated in bouts of 10 min or more) for 28 (± 31) min/day. According to the sleep diary, their nighttime sleep and daytime naps lasted for an average of 7.8 (±1.3) h/day and 10 (±16) min/day, respectively. Reported daytime naps were not included in the sleep parameters that entered further analysis.

The waking wear time compliance with the ActiGraph was 15.6 ± 2.2 h/day (i.e., 98.3 ± 5.0% of reported wake time). The wear time compliance with the Polar Vantage M, Garmin Vivoactive 4s and Garmin Vivosport was as high as 24.0 ± 0.1 h/day, 23.9 ± 0.5 h/day and 23.9 ± 0.5 h/day, respectively.

#### 3.2.1. Validity Evaluated

**Figure 4:**
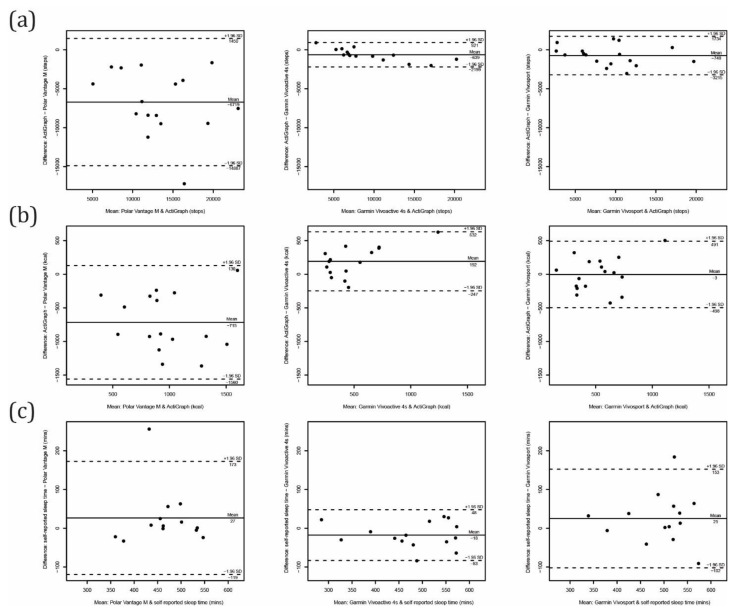
Bland–Altman plots for free-living conditions: (**a**) steps counted, (**b**) active calories burned and (**c**) sleep time.

**Figure 5:**
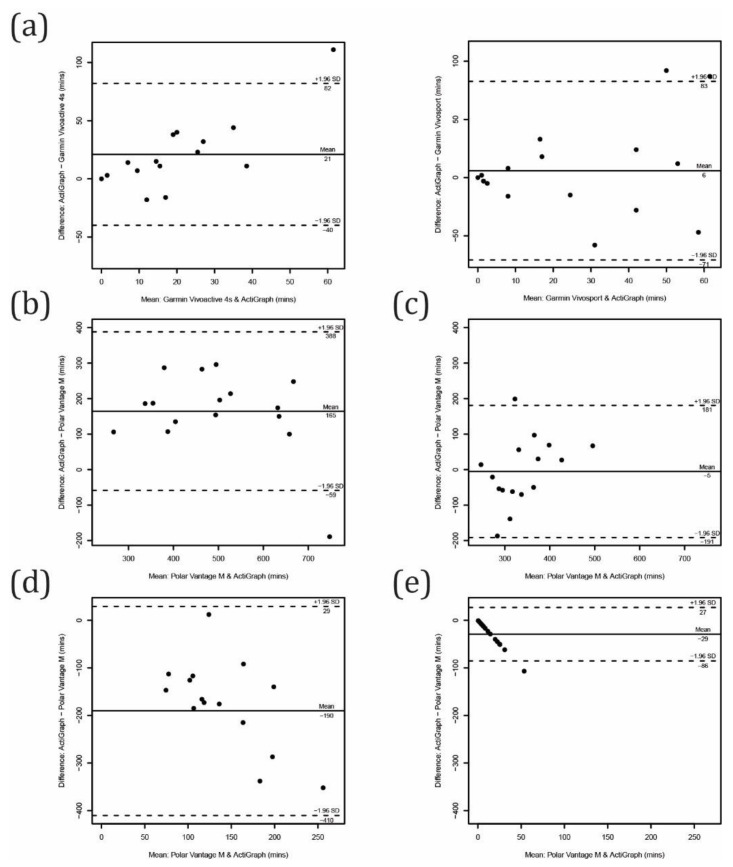
Bland–Altman plots for free-living conditions: (**a**) intensity minutes, (**b**) sedentary behavior, (**c**) light physical activity, (**d**) moderate physical activity and (**e**) vigorous physical activity.

The comparisons between computed activity parameters for average values of at least three valid days for each activity tracker and the ActiGraph are shown in Table 3 and Figure 4 and Figure 5. Comparing the step count, all consumer-grade devices significantly overestimated the number of steps taken. The overestimation was much higher for the Polar Vantage M (6719 ± 4168 steps) than for the Garmin Vivosport (740 ± 1262 steps) and Vivoactive 4s (639 ± 796 steps). The latter showed the best accuracy at the group level with a MPE of 5%, and at the individual level with a MAPE of 9%. Additionally, with the standard deviation of the difference (random error) of 796 steps/day and limits of agreement from Bland–Altman ranging from −2199 to 921 steps/day, the Garmin Vivoactive 4s showed the best precision. The ICC_2,1_ showed excellent agreement (0.98–0.95, *p* = 0.000) for both the Garmin devices, and poor agreement for the Polar device (0.38, *p* = 0.001).

When evaluating active calories, there was a systematical overestimation from the Polar Vantage M (with a MPE of 201% and MAPE of 202%), and underestimation from the Garmin Vivoactive 4s (with MPE of 26% and MAPE of 40%). The agreement for evaluating active calories was fair for the Garmin devices (ICC_2,1_ of 0.55 for Vivosmart and 0.58 for Vivoactive 4s), and poor for the Polar device (ICC_2,1_ of 0.15).

Comparing computed intensity minutes from the Garmin devices with the bouted MVPA obtained by the ActiGraph revealed a low level of agreement (ICC_2,1_ = 0.01 to 0.14). The Garmin activity trackers may not be reliable in estimating the bouted MVPA in older adults. Similarly, time spent in moderate and in vigorous PA from Polar Vantage M showed poor performance (ICC_2,1_ = −0.01 to 0.00), while the agreement for LIPA (ICC_2,1_ = 0.30) and for SB (ICC_2,1_ = 0.39) was somewhat better but still categorized as poor. Time spent in SB was significantly underestimated (by an average of 2.8 h/day) with a MPE of 30% and MAPE of 33%. LIPA was somewhat underestimated, and the MPE (8%) showed reasonable accuracy on the group level.

The performance for evaluating sleep time (when compared with self-reported sleep time) was much better, with MAPE ranging from 7% to 9% for all devices tested. The Polar Vantage M and Garmin Vivosport underestimated the sleep time by an average of 27 min/day and 25 min/day, respectively, while the Garmin Vivoactive 4s overestimated by 18 min/day. Limits of agreement from Bland–Altman revealed the best precision for the Garmin Vivoactive 4s (ranging from −83 to 48 min/day). The ICC_2,1_ also showed excellent agreement for the Garmin Vivoactive 4s (ICC_2,1_ = 0.93) and good agreement for the Garmin Vivosport (ICC_2,1_ = 0.61) and Polar Vantage M (ICC_2,1_ = 0.62).

#### 3.2.2. Evaluated Reliability and Sensitivity 

The reliability and sensitivity calculations for single-day activity outputs from all devices are shown in Table 4, and for an average of three consecutive days in Table 5. The ANOVA results revealed that no systematic differences between the multiple days of monitoring were present. The relative and absolute reliability and MDC were generally better for average day outputs (Table 5) when compared to single-day outputs (Table 4). However, the latter was not the case for relative reliability for steps, calories and intensity minutes from the Garmin Vivoactive 4s. The results for the average day were based only on seven participants, and we believe that the reason for compromised results is the small sample size. An ICC_2,1_ of at least 0.8 was often proposed as sufficient for the metric to be considered reliable [30]. None of the single-day measures reached that threshold and only some of the average day measures from the Polar Vantage M, Garmin Vivosport and ActiGraph satisfied the criteria; indicating that three days of measurements may not be enough to reliably capture all the computed variables (on the individual level).

The MDC for average steps ranged from 5646 to 6987 for the ActiGraph and Garmin Vivosport, respectively, and for average active calories burned it ranged from 289 to 380 for the Garmin Vivoactive 4s and Vivosport, respectively. These values of MDC are almost as high as the observed averages of steps taken and calories burned, indicating that a large change on the individual level needs to be observed so it can be attributed to a real change. The ActiGraph did not perform much better than the tested consumer-based activity trackers for those metrics. The MDC for average and single-day SB and PA outputs from the Polar Vantage M were only slightly compromised when compared with the ActiGraph. Average intensity minutes from the Garmin devices even showed a lower MDC than the bouted MVPA from the ActiGraph. For sleep parameters, the MDC was the lowest for the Polar Vantage M (66 min) and the highest for the Garmins (125 min).

Since no differences between multiple days were observed, responsiveness coefficients (ES and GR) could only be interpreted in terms of comparisons between computed outputs from different devices (e.g., which device provided more/less responsive metrics). Responsiveness for within-subject observations (ES) for steps count was shown to be largest for a single-day measure from the Garmin Vivoactive 4s (ES = 0.206) and ActiGraph (ES = 0.174), and for an average day measure for the Garmin Vivosport (ES = 0.174). The Garmin Vivoactive 4s also performed best for active calories burned for a single day (ES = 0.193) and average day (ES = 0.718). The single-day SB and LIPA metrics from Polar showed slightly better within-subject responsiveness than the ActiGraph, while for the average day, the ActiGraph’s SB and VIPA were shown to be more responsive. Intensity minutes were shown to be most responsive when measuring with the Garmin Vivosport (ES = 0.516 and 0.208 for a single day and the average day, respectively). The latest also showed the best responsiveness for sleep for single-day measures (ES = 0.671), while for an average day, the Polar Vantage M performed best (ES = 0.371).

When responsiveness for between-subject observations (GR) were of interest, the Garmin Vivoactive 4s (GR = 0.411) and Actigraph (GR = 0.307) performed best for single-day step counts, and the Garmin Vivosport (GR = 0.288) performed best for the average day. For active calories, both Garmin devices showed high responsiveness (GR = 0.933 and 0.536 for the Vivosport and Vivoactive 4s, respectively) for the average-day measures, while for a single day, the ActiGraph performed best (GR = 0.302). SB and PA outputs computed by the Actigraph were only slightly more responsive compared with those from Polar Vantage M, while intensity minutes from the Garmin Vivosport showed slightly higher responsiveness than the bouted MVPA from the ActiGraph. The Garmin Vivosport showed best responsiveness (GR = 1.302) for single-day sleep time measures, and Polar Vantage M (GR = 0.963) was the best in case of the average day. The Garmin Vivoactive 4s and the diary-reported sleep time showed similar responsiveness, which was higher for the average day (GR = 0.422 and 0.422) compared with single-day measures (GR = 0.221 for self-reported and GR = 0.194 for Garmin Vivoactive 4s).

## 4. Discussion

This was a comprehensive evaluation of validity, reliability and sensitivity to detect changes of several computed metrics from three popular consumer-based activity trackers (Polar Vantage M, Garmin Vivoactive 4s and Garmin Vivosport) when worn by older adults. The results showed excellent performance for measuring steps in controlled and free-living conditions for the Garmin Vivoactive 4s and Garmin Vivosport, while the Polar Vantage M consistently overestimated steps. The tested activity trackers showed good-to-excellent agreement for measuring sleep time, and poor-to-fair agreement for active calories. Polar Vantage M showed fair agreement for SB and LIPA when compared with the ActiGraph, while intensity minutes from both Garmin devices showed very poor agreement. MDC was consistently lower for averaged measures from three days compared to single-day measures, while responsiveness for single-day measures was generally higher for step counts, lower for sleep and mixed for all other computed variables.

### 4.1. Comparisons to Previous Studies

To the best of our knowledge, this was the first study to examine the validity of the Polar Vantage M, Garmin Vivoactive 4s and Garmin Vivosport when worn by older adults. Previous studies explored the validity of energy expenditure [31,32] and HR [32], as well as test-retest reliability [33] for Polar Vantage M under controlled laboratory conditions, while including young healthy adults only. When measuring energy expenditure, the results revealed mixed findings; Gilgen-Ammann et al. reported high accuracy for sedentary activity (MAPE = 1.2%) and walking (MAPE = 9.0%), and somewhat compromised accuracy for several types of physical activities tested (MAPE = 11.8% to 27.5%), while Düking et al. reported a very large standardized typical error of the estimate for sitting and walking (sTEE = 1.33 and 1.10, respectively), a large error for vigorous running (sTEE = 0.97–0.65) and a moderate error (sTEE = 0.34–0.32) for very vigorous running. While both studies used the same criterion instrument (Metamax 3B) and selected activities were performed during a monitoring period of similar length (5–10 min for each activity), the studies differ by sub-models of the tracker tested (Polar Vantage M for Gilgen-Ammann et al. and Polar Vantage V for Düking et al.) and training mode selected to monitor specific activity. Gilgen-Ammann et al. selected “other indoor” mode for sedentary activity, “walking” mode for walking, “running” mode for running, etc., while Düking et al. selected “running (treadmill)” mode for all activities tested including for sitting. Whether the discrepancy between the results of those two studies could be attributed to different sub-models of the Polar Vantage or to the specific mode selected is not known. In our study, no training mode was selected, since participants wore the device for several days continuously. Nevertheless, the Polar computed a daily summary of the metrics and included total calories burned. After calories attributed to BMR were deducted, the calculated active calories burned were compared with calories computed by the ActiGraph. The Polar Vantage M consistently overestimated calories burned (for an average of 780 kcal/day) and the agreement with the ActiGraph was very poor (ICC_2,1_ = 0.16). The previous findings suggested that the performance of Polar Vantage M for estimating energy expenditure could be reasonably good if the proper mode of activity is chosen, while our results revealed that its performance for the daily estimate of calories burned is poor (at least for older adults).

For the Garmin Vivoactive 4s, previous studies evaluated the validity of the step count, covered distance and energy expenditure during walking/running protocol [34] and HR and distance covered during structured outdoor protocol [35], both including healthy adults. No validity study for the Garmin Vivosport was found. Wahl et al. reported that the performance of the Garmin Vivoactive 4s for the step count is excellent, with the MAPE within 1% for brisk walking and different running speeds. For energy expenditure, the MAPE falls within 10% for brisk walking and high-speed running, while it was as high as 37% for slow-to-intermediate running speed. No significant under- or overestimation was reported. The results for the step count are in line with our findings; the Garmin Vivoactive 4s showed excellent accuracy, with a MAPE of 2% during preferred pace and slow pace walking in controlled conditions, and a MAPE of 9% during free-living conditions when worn by older adults. However, household chores, like tidying the dish as in our study, present a major challenge in capturing steps accurately, since there are substantial arm movements while not many steps are taken. During the 5 min of dish handling, the MAPE exceeds 100%, although it should be highlighted that the Garmin Vivoactive 4s performed best among the activity trackers tested in our study. Similarly, it performed best also during the cards task where the Garmin Vivoactive 4s erroneously counted some steps in only 25% of participants, while this happened in the Garmin Vivosport in 36% and the Polar Vantage M in 89% of participants, despite the fact that the activity tracker was worn on a non-dominant wrist. These findings are in line with previous studies that reported the stationary activity with substantial arm movement is challenging for wrist-worn trackers to capture accurately [36]. Additionally, in line with previous studies, performance of the Garmin Vivoactive 4s for estimating energy expenditure was compromised, as it turned out it significantly overestimated calories burned (for 192 kcal/day) when compared with the ActiGraph. The Garmin Vivosport showed accuracy on the group level (ME = −3 kcal/day), but not on the individual level (MAPE = 50%).

To date, several validation studies of wrist-worn consumer trackers have included the older adult population [13,15]. Most studies investigated steps and reported that the accuracy of measuring steps is generally high, but is deteriorated at slow speed walking [37,38] or when using walking aids [39,40], where steps taken are significantly underestimated. Phillips et al. reported a walking speed below 2 km/h was found to be challenging for the Fitbit Classic, and Thoroup et al. reported a speed of at least 3.6 km/h is needed for steps being counted with acceptable accuracy for the Fitbit Zip. In the older adult population with significantly compromised walking capacity, locations other than the wrist might perform better [37,41]. However, our sample of older adults had no problems with ambulation and the results obtained within the free-living study showed good performance for both Garmin devices (ICC_2,1_ = 0.95–0.98), but not for the Polar Vantage M device (ICC_2,1_ = 0.37), which significantly overestimated daily steps (for 6719 ± 4168 steps).

A few studies have also evaluated other daily activity metrics apart from steps. The performance of Fitbit (Flex, Charge HR and Charge 2) and Garmin (Vivosport HR+) devices for estimating MVPA was shown to be reasonably good; studies reported a moderate-to-good correlation with the criterion measure [20,42,43]. Alharbi et al. reported an overestimation of MVPA (for 10 min/day) using the Fitbit Flex, with a mean error of 10% in a population of older cardiac patients. Sensitivity and specificity to classify participants whether they adhere to PA guideline was reported to be high. In contrary, Tedesco et al. found the MAPE from the Fitbit Charge2 and Garmin Vivosmart HR+ to be as high as 76% and 100%, respectively. The authors proposed those two trackers may not be appropriate for MVPA estimates in older adults. Those findings are in line with ours, where a MAPE from the Garmin Vivoactive 4s and Vivosport of 117% and 291%, respectively, was found. It should be noted that Fitbit and Garmin counted only MVPA minutes that occurred in bouts of at least 10 min. Moreover, vigorous minutes are doubled when added to intensity minutes (i.e., MVPA) in Garmin devices. Those requirements are in line with previous PA guidelines [44], but current guidelines removed the 10 min bout criterion [45]. The Fitbit device responded to that change in guidelines quickly and introduced a new MVPA metric (“Active Zone Minutes”) in early 2020, resulting in “every minute counts” [46]. It is expected that other brands of trackers will also do something similar soon. Nevertheless, the bouted MVPA metric still has value in PA research and practice, since it relates to the health outcomes [47].

It is of vital importance that activity trackers compute movement behavior metrics that are highly relevant for health and well-being. Currently, a 24 h movement paradigm that highlights the importance of the whole spectrum of movement behaviors, including sleep, is gaining momentum [48,49,50]. Specifically, LIPA is of great importance for older adults [51], especially for those who might not have the capacity to engage in MVPA. To date, activity trackers mostly provide data on MVPA and sleep, but rarely on LIPA and SB. The Polar Vantage M device tested in our study computes metrics that cover the whole 24 h day; we found good agreement for sleep (ICC_2,1_ = 0.62), fair for SB and LIPA (ICC_2,1_ = 0.44 and 0.45 for single-day measures, respectively) and poor for MVPA (ICC_2,1_ ~ 0.00), when compared with ActiGraph. Similarly, Boeselt et al. [52] reported poor correlation (r = 0.25) for daily activity time (a sum of LIPA and MVPA) from the Polar A300, and Collins et al. found an overestimation of SB for 37% and underestimation of MVPA for 50% for the Fitbit Charge2. For sleep time, the Fitbit Flex and Fitbit Charge HR showed to correlate poorly with the criterion (rho = 0.37) [20], while moderate agreement (ICC_2,1_ = 0.67) was found for the Fitbit Charge2, and poor agreement (ICC_2,1_ = 0.42) for the Garmin Vivosmart HR+ [42]. In contrast, we found excellent agreement for the Garmin Vivoactive 4s (ICC_2,1_ = 0.93) and good agreement for the Garmin Vivosport (ICC_2,1_ = 0.60). Moreover, all tested trackers showed good performance for measuring sleep on the individual level, with a MAPE below 10%. It might be the case that proprietary algorithms for detecting sleep improved importantly over the past years [53]. Taken together, a certain level of accuracy could be observed for most of the computed movement behaviors metrics in the older adult population. Studies mainly included healthy older adults and future validation studies should include also those with mobility limitations. Despite accuracy of the measure is of vital importance, other measurement properties, like sensitivity to detect change, are crucial for some scenarios, which are discussed below.

### 4.2. Implications for Clinical and Research Purposes

Currently, there is no consensus about clear-cut standards for validity, reliability and sensitivity metrics that would indicate a certain computed metric is adequate to be used for a specific situation. However, it has been proposed that for research purposes, the acceptable MAPE under controlled conditions is 3% and under free-living conditions is 10%, and that the acceptable MAPE for clinical purposes under free-living conditions is 20% [13]. Applying these criteria, steps from the Garmin Vivoactive 4s could be used for research and clinical purposes, while from the Garmin Vivosport, they could only for clinical purposes. All the consumer-based trackers could be used to monitor sleep time for both purposes, whereas all other computed variables present a MAPE of more than 20%. However, we believe such simplification could be misleading and metrics with a measurement error far beyond 20% still hold value for certain research or clinical purposes. For example, the majority of current knowledge about health benefits of PA comes from studies that used self-reported measures, which possess a substantial measurement error [54]. For epidemiological studies on associations between movement behaviors and health, it may be most crucial that movement behavior metrics correlate with the true value. Applying these criteria, all measures, except intensity minutes from the Garmin devices and the calories burned and MVPA from the Polar device, could be used for the epidemiological research.

In clinical settings or mHealth applications, the change in behavior on the individual level is often of interest. Behavior naturally varies between days and activity trackers come with some measurement error—both factors raise a question about what change needs to be observed that could be interpreted as a real change in the behavior. The MDC provides the answer; it was consistently higher for single-day measures than for average-day measures, which is in line with previous findings [12]. By averaging multiple days, the variability of the score and consequently the MDC is lowered. When averaging three days as in our study, the MDC was still relatively high for all outputs, and further research is needed to evaluate the effect of capturing longer periods on MDC. In terms of responsiveness of the tested metrics, our results presented mixed findings regarding the period duration (e.g., single-day or average-day metrics) and the devices tested. We observed a trend where the most responsive specific metric obtained by a certain device showed to be the most responsive according to both (ES and GR) coefficients (e.g., a single-day SB metric obtained by the Polar Vantage M was the most responsive for within-subject behavior (ES) and between-subject behavior (GR) change on the group level). In general, the research-grade monitor ActiGraph did not perform any better than consumer-based trackers regarding responsiveness. This finding indicates that the three consumer-based trackers tested have a similar ability to detect changes in longitudinal and clinical trial research than the ActiGraph. However, it should be highlighted that validity of consumer-based trackers is somewhat compromised for most outputs, while completely absent for the others. In the latter case, the use of such metric is not advised, regardless of if the responsiveness coefficients are shown to be high. Indeed, it is possible that a metric is reliable and responsive, but not valid (i.e., it does not measure what it is supposed to measure).

Despite promising measurement properties of some output metrics, when working with consumer-based activity trackers, several limitations should be considered. Garmin and Polar currently do not offer batch downloading (Polar does not offer any downloading of daily metrics), which presents a substantial burden for researchers. Additionally, data history needs to be deleted before a new participant starts to wear the device, and the participant’s personal information needs to be entered manually, since this affects calculations of the output metrics. Moreover, a researcher or a practitioner does not have clear information about the non-wear time in Garmin, which is a critical issue affecting the validity of the data. Only computed daily summary metrics are available, while raw minute-by-minute data or proprietary algorithms that computed the metrics are not available. Updates of proprietary applications/algorithms are released regularly, which might affect the way data are being processed and consequently affect the measurement properties of computed metrics. Many models of activity trackers (including Garmin) are computing “outdated” health metrics, like counting only MVPA periods that are longer than 10 min. Lastly, there are issues related to data privacy and the ownership of the data; thus, more work is needed to resolve this uncertainty.

### 4.3. Strengths and Limitations

The main strength of our study is the comprehensive approach to evaluating the measurement properties of the three popular activity trackers in controlled conditions while mimicking common everyday activities, as well as free-living conditions, which is the most ecologically valid environment. However, our results should be interpreted in light of several limitations. Firstly, findings about waking movement behaviors could not be generalized to the older adult population with physical impairments that significantly affect ambulation (e.g., using rollator, walking stick or walking extremely slowly), and findings about sleep metrics are limited to the population free of substantial sleep disturbances. However, our results could be generalized to a generally healthy older adult population, since the diversity of our sample was substantial; the age ranged from 60 to 84 years, the BMI ranged from 20 to 39 and PA levels ranged from accumulating on average 2670 to 18,833 steps/day. Secondly, the sample sizes were generally small, especially for the average day reliability and sensitivity to change for the Garmin Vivoactive 4s. The latter should be interpreted with great caution. Thirdly, in the first part of the study (controlled conditions), the position of the activity trackers on the wrist was not randomized between the participants. Fourthly, the validity indicators for tested consumer-based activity trackers were calculated while using data captured by the research-grade monitor ActiGraph as a convergent measure. It should be highlighted that the ActiGraph also suffers from its own validity limitations, although it is a well-established research device that provides reasonably valid estimates on movement behaviors across the whole activity spectrum [55]. Finally, activity trackers require defining the level of user’s physical activity/fitness, which might affect the computed outputs (for all participants we chose the lowest training background; for the Polar device it was the “occasional training background” and for the Garmin devices it was “occasional, light exercise” activity class).

## 5. Conclusions

To the best of our knowledge, this was the first study to examine the validity of the Polar Vantage M, Garmin Vivoactive 4s and Garmin Vivosport in older adults, and one of the few studies that compared sensitivity to detect changes between commercially available wrist-worn activity trackers and a research-grade physical activity monitor. Our comprehensive evaluation of the measurement properties revealed that step count, active calories burned and sleep time metrics obtained with the Garmin Vivoactive 4s and Garmin Vivosport showed acceptable agreement when compared with a research-grade monitor when worn by older adults. For the Polar Vantage M, acceptable agreement was found for SB, LIPA and sleep time metrics. However, it should be highlighted that a different balance between validity, reliability and sensitivity to detect changes as well as the length of the monitoring period is needed for different purposes—for individual use/care, longitudinal settings or clinical trial settings. The results provided in this study, as well as some other practical and legal considerations noted, could be used to guide choices on the usage of activity trackers tested in research or clinical setting.

## Figures and Tables

**Table 1:** Participant’s characteristics.

	Participants, n	Age (SD), Years	Body Height (SD), cm	Body Mass (SD), kg	BMI (SD), kg/m^2^
**Controlled conditions**					
male	13	74.7 (4.6)	176 (8)	79.5 (8.3)	25.7 (2.0)
female	15	74.0 (5.3)	164 (6)	69.4 (7.7)	25.8 (3.1)
all	28	74.3 (4.9)	169 (9)	74.1 (9.3)	25.8 (2.6)
**Free-living conditions**					
*Polar Vantage M*					
male	7	69.9 (5.2)	179 (5)	82.2 (9.2)	25.7 (2.7)
female	9	68.8 (5.8)	160 (4)	66.9 (9.3)	26.0 (2.9)
all	16	69.3 (5.4)	168 (11)	73.6 (11.9)	25.9 (2.7)
*Garmin Vivoactive 4s*					
male	5	70.7 (2.8)	174 (6)	76.6 (8.8)	25.2 (2.5)
female	12	69.2 (6.3)	162 (6)	71.3 (13.9)	27.3 (5.1)
all	17	69.6 (5.5)	166 (9)	72.9 (12.6)	26.6 (4.6)
*Garmin Vivosport*					
male	7	73.6 (5.8)	175 (10)	75.5 (6.6)	24.6 (1.7)
female	10	70.9 (8.1)	160 (3)	68.9 (15.8)	27.0 (5.8)
all	17	72.0 (7.2)	166 (10)	71.6 (13.0)	26.0 (4.7)

Abbreviations: SD, standard deviation; F, female; BMI, body mass index.

**Table 2:** Calculated mean steps, indicators of error and agreement for steps from each activity tracker/task tested in controlled conditions (n = 28). Actual steps taken (counted visually by a researcher) were used as a criterion measure.

	Mean (SD), Steps	ME (SD), Steps	MPE (SD), %	MAPE (SD), %	RMSE (SD), Steps	ICC_2,1_	*t*-Test (*p*-Value)
Preferred pace walking							
Polar Vantage M	575 (66)	12 (41)	2 (8)	6 (6)	42 (60)	0.75 (*p* = 0.000)	0.139
Garmin Vivoactive 4s	582 (53)	5 (16)	1 (3)	2 (2)	17 (25)	0.95 (*p* = 0.000)	0.138
Garmin Vivosport	590 (59)	−3 (23)	−1 (4)	2 (4)	23 (48)	0.92 (*p* = 0.000)	0.452
Slow pace walking							
Polar Vantage M	482 (84)	27 (77)	5 (16)	11 (13)	80 (122)	0.37 (*p* = 0.019)	0.075
Garmin Vivoactive 4s	501 (49)	8 (23)	2 (4)	2 (4)	24 (40)	0.89 (*p* = 0.000)	0.062
Garmin Vivosport	505 (50)	4 (15)	1 (3)	2 (2)	15 (29)	0.96 (*p* = 0.000)	0.158
Tidying the dish							
Polar Vantage M	282 (119)	−240 (122)	−751 (484)	751 (484)	268 (254)	0.00 (*p* = 0.524)	0.000
Garmin Vivoactive 4s	45 (35)	−3 (47)	−49 (141)	107 (102)	46 (56)	−0.18 (*p* = 0.820)	0.702
Garmin Vivosport	79 (41)	−37 (51)	−147 (175)	160 (163)	62 (67)	−0.10 (*p* = 0.795)	0.001
Playing cards							
Polar Vantage M	47 (91)	−47 (91)	NaN	NaN	101 (180)	NaN	0.011
Garmin Vivoactive 4s	7 (14)	−7 (14)	NaN	NaN	16 (25)	NaN	0.015
Garmin Vivosport	23 (49)	−23 (49)	NaN	NaN	53 (98)	NaN	0.020
All controlled tasks							
Polar Vantage M	1386 (236)	−248 (212)	−22 (20)	23 (18)	324 (368)	0.18 (*p* = 0.035)	0.000
Garmin Vivoactive 4s	1135 (102)	2 (55)	0 (5)	3 (3)	54 (80)	0.87 (*p* = 0.000)	0.813
Garmin Vivosport	1196 (119)	−59 (65)	−5 (6)	6 (5)	87 (111)	0.75 (*p* = 0.000)	0.000

Abbreviations: SD, standard deviation; ME, mean error; MPE, mean percentage error; MAPE, mean average percentage error; RMSE, root mean square error; LoA, limits of agreement.

**Table 3:** Calculated means, indicators of error and agreement for each computed activity output (of an average of at least three valid days) from each activity tracker in free-living conditions. ActiGraph with self-reported sleep time was used as a convergent measure.

	Mean (SD), Steps	ME (SD), Steps	MPE (SD), %	MAPE (SD), %	RMSE (SD), Steps	ICC_2,1_	*t*-Test (*p*-Value)
Polar Vantage M (n = 16)							
steps	16735 (5690)	−6719 (4168)	−84 (63)	84 (63)	7838 (8547)	0.37 (*p* = 0.001)	0.000
active kcal	1328 (412)	−715 (431)	−201 (228)	202 (228)	828 (783)	0.15 (*p* = 0.056)	0.000
SB (mins)	415 (168)	165 (114)	30 (19)	33 (11)	198 (160)	0.39 (*p* = 0.000)	0.000
LIPA (mins)	342 (62)	−5 (95)	−8 (34)	25 (24)	92 (110)	0.30 (*p* = 0.132)	0.844
MIPA (mins)	243 (102)	−191 (112)	−1407 (2987)	1408 (2987)	219 (229)	-0.01 (*p* = 0.539)	0.000
VIPA (mins)	29 (29)	−29 (29)	NaN	NaN	40 (54)	0.00 (*p* = 0.500)	0.001
sleep (mins)	437 (81)	27 (75)	5 (14)	8 (12)	76 (134)	0.62 (*p* = 0.004)	0.121
Garmin Vivoactive 4s (n = 15)							
steps	9726 (5113)	−639 (796)	−5 (11)	9 (6)	1000 (1116)	0.98 (*p* = 0.000)	0.006
active kcal	389 (212)	192 (224)	26 (36)	40 (19)	290 (324)	0.58 (*p* = 0.001)	0.005
int mins	10 (10)	21 (31)	−23 (201)	117 (162)	37 (55)	0.01 (*p* = 0.473)	0.020
sleep (mins)	490 (93)	−18 (33)	−4 (7)	7 (4)	37 (43)	0.93 (*p* = 0.000)	0.116
Garmin Vivosport (n = 17)							
steps	9568 (4843)	−740 (1262)	−8 (16)	15 (9)	1431 (1571)	0.95 (*p* = 0.000)	0.028
active kcal	526 (225)	−3 (252)	−19 (64)	50 (43)	245 (267)	0.55 (*p* = 0.011)	0.958
int mins	22 (25)	6 (39)	−201 (809)	291 (779)	39 (51)	0.14 (*p* = 0.300)	0.540
sleep (mins)	479 (72)	25 (65)	4 (12)	9 (8)	67 (94)	0.60 (*p* = 0.005)	0.161

Abbreviations: SD, standard deviation; ME, mean error; MPE, mean percentage error; MAPE, mean average percentage error; RMSE, root mean square error; LoA, limits of agreement; SB, sedentary behavior; LIPA, low-intensity physical activity; MIPA, moderate-intensity physical activity; VIPA, vigorous-intensity physical activity; int min, intensity minutes.

**Table 4:** Absolute and relative reliability, minimal detectable change and responsiveness of computed activity outputs (of a randomly selected single day) from each activity tracker and ActiGraph in free-living conditions.

	Mean (SD), Random Day 1	Mean (SD), Random Day 2	Systematic Difference	ICC_2,1_ (95% CI)	SEM	MDC	ES	GR
Polar Vantage M (n = 16)								
steps	16,330	16822	F (2,32) = 0.086,	0.68	3908	10,832	0.077	0.126
	(6358)	(6941)	*p* = 0.918, ηp2 = 0.005	(0.43, 0.85)				
active kcal	1330	1380	F (2,32) = 0.000,	0.80	215	597	0.099	0.232
	(503)	(496)	*p* > 0.999, ηp2 = 0.000	(0.61, 0.91)				
SB (mins)	384	371	F (2,30) = 0.196,	0.66	83	229	0.087	0.157
	(149)	(141)	*p* = 0.823, ηp2 = 0.013	(0.39, 0.85)				
LIPA (mins)	353	364	F (2,32) = 0.700,	0.43	68	187	0.169	0.163
	(65)	(99)	*p* = 0.504, ηp2 = 0.042	(0.13, 0.71)				
MIPA (mins)	247	252	F (2,32) = 0.432,	0.75	54	149	0.043	0.093
	(117)	(105)	*p* = 0.653, ηp2 = 0.026	(0.54, 0.89)				
VIPA (mins)	28	30	F (2,32) = 0.072,	0.74	19	53	0.054	0.105
	(37)	(44)	*p* = 0.931, ηp2 = 0.004	(0.51, 0.88)				
sleep (mins)	430	419	F (2,30) = 0.002,	0.42	79	218	0.128	0.140
	(86)	(129)	*p* = 0.998, ηp2 = 0.000	(0.11, 0.71)				
Garmin Vivoactive 4s (n = 16)								
steps	9300	10,444	F (2,28) = 0.332,	0.70	2783	7714	0.206	0.411
	(5557)	(3979)	*p* = 0.720, ηp2 = 0.023	(0.44, 0.88)				
active kcal	370	412	F (2,28) = 0.584,	0.66	161	446	0.193	0.261
	(218)	(200)	*p* = 0.564, ηp2 = 0.040	(0.38, 0.85)				
int mins	8	9	F (2,28) = 0.564,	0.24	15	42	0.056	0.065
	(18)	(18)	*p* = 0.575, ηp2 = 0.039	(−0.06, 0.59)				
sleep (mins)	507	490	F (2,28) = 0.946,	0.41	87	242	0.189	0.194
	(90)	(115)	*p* = 0.401, ηp2 = 0.063	(0.09, 0.71)				
Garmin Vivosport (n = 17)								
steps	10,766	10,841	F (2,32) = 0.208,	0.65	3461	9592	0.011	0.022
	(6732)	(6134)	*p* = 0.814, ηp2 = 0.013	(0.39, 0.84)				
active kcal	606	591	F (2,32) = 2.560,	0.48	206	572	0.045	0.073
	(337)	(370)	*p* = 0.093, ηp2 = 0.138	(0.18, 0.74)				
int mins	21	37	F (1.4,23.1) = 0.256,	0.19	41	113	0.516	0.391
	(31)	(68)	*p* = 0.703, ηp2 = 0.016	(−0.09, 0.53)				
sleep (mins)	501	452	F (2,28) = 2.985,	0.74	38	104	0.671	1.302
	(73)	(101)	*p* = 0.067, ηp2 = 0.176	(0.51, 0.90)				
ActiGraph (n = 40)								
steps	9366	10316	F (2,102) = 1.048,	0.58	3091	8568	0.174	0.307
	(5456)	(5632)	*p* = 0.355, ηp2 = 0.020	(0.40, 0.73)				
active kcal	586	643	F (2,102) = 2.054,	0.67	189	524	0.153	0.302
	(373)	(408)	*p* = 0.133, ηp2 = 0.039	(0.52, 0.80)				
SB (mins)	579	574	F (2,102) = 1.695,	0.65	78	215	0.035	0.064
	(144)	(133)	*p* = 0.189, ηp2 = 0.032	(0.49, 0.78)				
LIPA (mins)	316	324	F (2,102) = 1.003,	0.64	60	167	0.085	0.132
	(94)	(106)	*p* = 0.370, ηp2 = 0.019	(0.48, 0.77)				
MIPA (mins)	50	56	F (1.8,89.4) = 0.830,	0.61	29	81	0.122	0.204
	(49)	(48)	*p* = 0.426, ηp2 = 0.016	(0.44, 0.75)				
VIPA (mins)	1	1	F (1.0,52.1) = 1.878,	0.02	7	19	0.000	0.000
	(7)	(6)	*p* = 0.176, ηp2 = 0.036	(−0.14, 0.22)				
bouted MVPA	30	38	F (2,102) = 0.760,	0.63	26	71	0.186	0.310
(mins)	(43)	(44)	*p* = 0.470, ηp2 = 0.015	(0.46, 0.76)				
Diary (n = 40)								
sleep (mins)	494	484	F (2,102) = 0.218,	0.75	45	125	0.120	0.221
	(83)	(85)	*p* = 0.805, ηp2 = 0.004	(0.62, 0.85)				

Abbreviations: SD, standard deviation; SEM, standard error of measurement; MDC, minimal detectable change; ES, Cohen’s effect size; GR, Guyatt’s responsiveness coefficient; SB, sedentary behavior; LIPA, low-intensity physical activity; MIPA, moderate-intensity physical activity; VIPA, vigorous-intensity physical activity; int min, intensity minutes.

**Table 5:** Absolute and relative reliability, minimal detectable change and responsiveness of computed activity outputs (of an average of three valid days) from each activity tracker and ActiGraph in free-living conditions.

	Mean (SD), Day 1–3	Mean (SD), Day 4–6	Systematic Difference	ICC_2,1_ (95% CI)	SEM	MDC	ES	GR
Polar Vantage M (n = 12)								
steps	14453	14320	F (1,11) = 0.021,	0.82	2229	6178	0.028	0.060
	(4668)	(5665)	*p* = 0.887, ηp2 = 0.002	(0.48, 0.94)				
active kcal	1365	1398	F (1,11) = 0.361,	0.86	133	368	0.102	0.248
	(322)	(393)	*p* = 0.560, ηp2 = 0.032	(0.59, 0.96)				
SB (mins)	427	430	F (1,10) = 0.027,	0.78	53	147	0.029	0.056
	(102)	(124)	*p* = 0.873, ηp2 = 0.003	(0.37, 0.94)				
LIPA (mins)	327	346	F (1,11) = 1.099,	0.43	43	120	0.345	0.440
	(55)	(59)	*p* = 0.317, ηp2 = 0.091	(−0.16, 0.79)				
MIPA (mins)	221	222	F (1,11) = 0.002,	0.90	33	91	0.010	0.030
	(100)	(106)	*p* = 0.966, ηp2 = 0.000	(0.69, 0.97)				
VIPA (mins)	28	26	F (1,11) = 0.132,	0.83	12	34	0.077	0.162
	(26)	(34)	*p* = 0.723, ηp2 = 0.012	(0.52, 0.95)				
sleep (mins)	411	434	F (1,8) = 4.217,	0.75	24	66	0.371	0.963
	(62)	(63)	*p* = 0.074, ηp2 = 0.345	(0.230, 0.94)				
Garmin Vivoactive 4s (n = 7)								
steps	7695	7781	F (1,6) = 0.006,	0.24	2116	5864	0.053	0.041
	(1608)	(3029)	*p* = 0.942, ηp2 = 0.001	(−0.56, 0.81)				
active kcal	311	367	F (1,6) = 0.981,	0.54	104	289	0.718	0.536
	(78)	(205)	*p* = 0.360, ηp2 = 0.140	(−0.26, 0.90)				
int mins	9	10	F (1,6) = 0.001,	0.06	11	31	0.083	0.090
	(12)	(11)	*p* = 0.982, ηp2 = 0.000	(−0.68, 0.73)				
sleep (mins)	482	501	F (1,5) = 0.030,	0.61	45	125	0.188	0.422
	(101)	(53)	*p* = 0.869, ηp2 = 0.006	(−0.27, 0.93)				
Garmin Vivosport (n = 11)								
steps	9327	8601	F (1,10) = 0.456,	0.66	2521	6987	0.174	0.288
	(4167)	(4418)	*p* = 0.515, ηp2 = 0.044	(0.13, 0.89)				
active kcal	623	495	F (1,10) = 4.815,	0.66	137	380	0.612	0.933
	(209)	(259)	*p* = 0.053, ηp2 = 0.325	(0.13, 0.90)				
int mins	18	23	F (1,10) = 1.107,	0.86	11	29	0.208	0.475
	(24)	(31)	*p* = 0.317, ηp2 = 0.100	(0.55, 0.96)				
sleep (mins)	474	464	F (1,9) = 1.131,	0.71	45	125	0.132	0.222
	(76)	(97)	*p* = 0.315, ηp2 = 0.112	(0.19, 0.92)				
ActiGraph (n = 39)								
Steps	8424	8468	F (1,38) = 0.008,	0.75	2037	5646	0.012	0.022
	(3696)	(4355)	*p* = 0.927, ηp2 = 0.000	(0.57, 0.86)				
active kcal	549	512	F (1,38) = 2.182,	0.87	109	303	0.120	0.338
	(309)	(303)	*p* = 0.148, ηp2 = 0.054	(0.77, 0.93)				
SB (mins)	624	632	F (1,38) = 0.381,	0.90	38	106	0.071	0.210
	(112)	(124)	*p* = 0.381, ηp2 = 0.020	(0.81, 0.94)				
LIPA (mins)	307	290	F (1,38) = 3.779,	0.82	38	104	0.202	0.452
	(84)	(93)	*p* = 0.059, ηp2 = 0.090	(0.68, 0.90)				
MIPA (mins)	44	44	F (1,38) = 0.008,	0.78	17	48	0.000	0.000
	(36)	(38)	*p* = 0.928, ηp2 = 0.000	(0.62, 0.88)				
VIPA (mins)	1	0	F (1,38) = 2.659,	0.42	2	6	0.250	0.436
	(4)	(1)	*p* = 0.111, ηp2 = 0.069	(0.11, 0.65)				
bouted MVPA	25	30	F (1,38) = 1.432,	0.76	16	45	0.167	0.311
(mins)	(30)	(35)	*p* = 0.239, ηp2 = 0.036	(0.59, 0.87)				
Diary (n = 39)								
sleep (mins)	462	475	F (1,38) = 3.998,	0.87	29	81	0.157	0.442
	(83)	(81)	*p* = 0.053, ηp2 = 0.095	(0.77, 0.93)				

Abbreviations: SD, standard deviation; SEM, standard error of measurement; MDC, minimal detectable change; ES, Cohen’s effect size; GR, Guyatt’s responsiveness coefficient; SB, sedentary behavior; LIPA, low-intensity physical activity; MIPA, moderate-intensity physical activity; VIPA, vigorous-intensity physical activity; int min, intensity minutes.

## Data Availability

The data presented in this study are openly available in repository Zenodo at doi.org/10.5281/zenodo.5158551 [56].

## Abbreviations

ANOVA	analysis of variance
BMR	basal metabolic rate
Cpm	counts per minute
ES	Cohen’s effect size
GR	Guyatt’s responsiveness coefficient
HR	heart rate
ICC	intraclass correlation coefficient
LIPA	light physical activity
MAPE	mean absolute percentage error
MDC	minimal detachable change
ME	mean error
MPE	mean percentage error
MVPA	moderate-to-vigorous physical activity
PA	physical activity
RMSE	root mean square error
SB	sedentary behavior
SD	standard deviation
SEM	standard error of measurement
sTEE	standardized typical error
